# Supplementary Tibial Fixation for Anterior Cruciate Ligament (ACL) Reconstruction Using Quadrupled Semitendinosus Graft: A Retrospective Study

**DOI:** 10.7759/cureus.84526

**Published:** 2025-05-21

**Authors:** Pankaj Aggarwal, Swagat Mahapatra, Mohd A Aslam, Vineet Kumar, Devashish Chhutani

**Affiliations:** 1 Orthopaedics and Traumatology, Dr. Ram Manohar Lohia Institute of Medical Sciences, Lucknow, IND

**Keywords:** acl reconstruction, ikdc, lysholm knee, standard fixation, supplementary fixation, tibial fixation

## Abstract

Background

Anterior cruciate ligament (ACL) reconstruction has seen significant advancements, but challenges remain, particularly in tibial fixation. Low bone density and prolonged osseous integration of soft tissue grafts compromise fixation, especially in the Indian population. We present a novel technique for supplementary ACL fixation using a transosseous tibial tunnel with a quadrupled semitendinosus graft, eliminating the need for additional hardware and associated complications.

Purpose

The purpose of this study was to compare clinical and functional outcomes of the addition of supplementary fixation with transosseous tibial tunnel with ongoing or prevailing standard bioscrew tibial fixation in ACL reconstruction surgeries using semitendinosus and gracilis graft.

Methodology

The study design was a retrospective review. Eighty-eight patients who underwent anterior cruciate ligament (ACL) reconstruction at our institution between 2019 and 2022 by the same surgeon and met the inclusion criteria were divided into two groups and were followed for 2 years postoperatively. The standard group, comprising 40 patients, underwent bioscrew for tibial fixation, and 48 patients of the supplementary fixation group underwent additional transosseous tibial tunnel fixation along with bioscrew. The outcomes, including Lysholm knee score, International Knee Documentation Committee (IKDC) score, Lachman test, Pivot score, Visual Analog Score (VAS), and thigh atrophy, were measured and compared at the final follow-up.

Results

Significant improvements were seen in the functional knee scores post-operatively in both groups, with significantly better results in the supplementary fixation group compared to the standard group for the Lysholm knee score, IKDC score, and Lachman test. The supplementary fixation group was also found to have better results for VAS, Pivot test, and thigh atrophy; however, the results were not significant.

Conclusion

Supplementary fixation with transosseous tibial tunnel is a safe, effective, and cost-efficient method for improving knee stability and clinical outcomes. The technique also eliminates the risk of symptomatic hardware complications, as was found in several previous studies, and avoids the need for extra implants, reducing overall treatment costs

## Introduction

Anterior cruciate ligament (ACL) ruptures are severe injuries that lead to chronic instability and degenerative changes in the knee joint [1]. The increasing incidence of high-energy road traffic accidents and sports injuries has resulted in a growing number of ACL tears across all age groups. Although arthroscopic ACL reconstruction is the standard treatment approach, debate continues regarding the optimal graft choice [2]. Both bone-patellar tendon-bone (BPTB) and hamstring grafts are commonly used, with the latter gaining popularity due to reduced graft site morbidity [2]. Hamstring grafts, specifically quadrupled semitendinosus or combined semitendinosus and gracilis grafts, have become a popular choice for ACL reconstruction [3,4]. While quadrupled semitendinosus grafts provide thicker grafts and preserve active knee flexion, they have shorter lengths compared to combined grafts [5-7]. Soft tissue grafts, such as hamstring grafts, require more time to heal compared to bone-tendon grafts and necessitate greater protection during the early post-operative phase [8]. When selecting a graft for ACL reconstruction, surgeons must weigh the benefits and drawbacks of each option, considering factors such as graft thickness, length, and morbidity.

Research has highlighted the importance of tibial fixation in ACL reconstruction, citing the distal femur's greater bone density compared to the proximal tibia. Additionally, the tibial tunnel's parallel orientation to the graft, in contrast to the femoral tunnel, underscores the need for secure tibial fixation [9]. The proximal tibial metaphysis's low bone density and the prolonged osseous integration period of soft tissue grafts (3-6 months) render tibial fixation vulnerable [10,11]. This vulnerability is further compounded by intensive rehabilitation protocols and the pressure to accelerate athletes' return to play, which can potentially compromise the graft's integrity. Inadequate tibial fixation leads to early failure and anterior-posterior laxity in the later period [12]. As a result, surgeons must prioritize secure tibial fixation to ensure successful ACL reconstruction and minimize the risk of complications. The growing trend of accelerated rehabilitation protocols has underscored the importance of secure tibial fixation, making it a pressing concern for surgeons [12].

Augmenting tibial fixation is crucial for secure graft fixation, especially with shorter quadrupled semitendinosus grafts. Accessory tibial fixation provides stronger initial fixation and reduced laxity, enabling aggressive rehabilitation and quicker return to sports. However, traditional methods using bone staples or tibial post screws have been associated with complications [12,13] such as anterior tibial tenderness, hardware-related pain, skin irritation, and wound complications.

We present a new technique for supplementary ACL fixation using a transosseous tibial tunnel for ACL reconstruction with quadrupled semitendinosus graft without the use of any extra hardware, thus avoiding the problems associated with symptomatic hardware.

## Materials and methods

Patient selection

A retrospective review was conducted on 98patients who underwent ACL reconstruction between 2019 and 2022. The retrospective study started in 2022. Follow-up results of operated patients were retrieved from hospital records. Patients operated in 2021-22 were further followed up until their 2-year follow-up was complete. Surgical data were retrieved from operative logs, and patients were followed up for 2 years postoperatively. The Institutional Ethics Committee of Dr. Ram Manohar Lohia Institute of Medical Sciences, Lucknow, issued approval IEC No. 204/22, and informed consent was obtained from all patients.

The primary surgeon initially performed standard ACL reconstruction using a quadrupled semitendinosus graft. However, due to concerns about the graft's length in the tibia, supplementary fixation using a transosseous tibial tunnel was added to all primary ACL reconstructions to provide additional strength and allow early rehabilitation.

Inclusion and exclusion criteria

The study included male and female patients aged 18-50 years with isolated primary ACL tears. Exclusion criteria included a knee with associated injuries or previous surgeries, abnormal knee conditions, semitendinosus graft length <28 mm, articular fractures or previous surgery on the same knee, and radiographic abnormalities. All patients underwent MRI and X-ray evaluations. The same surgeon performed all surgeries using a quadrupled semitendinosus graft with an adjustable loop Endobutton (Smith and Nephews, London, UK) for femoral fixation, with or without supplementary tibial tunnel fixation. Ten patients were lost to follow-up and excluded.

Two groups of patients underwent routine arthroscopic ACL reconstruction using hamstring grafts: 1) Standard Group (n=40), in which the patients received isolated bioscrew tibial fixation using a standard Smith & Nephew Endoscopy bioscrew (BIORCI HA screw, Smith and Nephews, London, UK) and 2) Supplementary Group (n=48), in which the patients received tibial bioscrew fixation with additional supplementary fixation using transosseous tibial fixation.

Surgical technique

**Figure 1:**
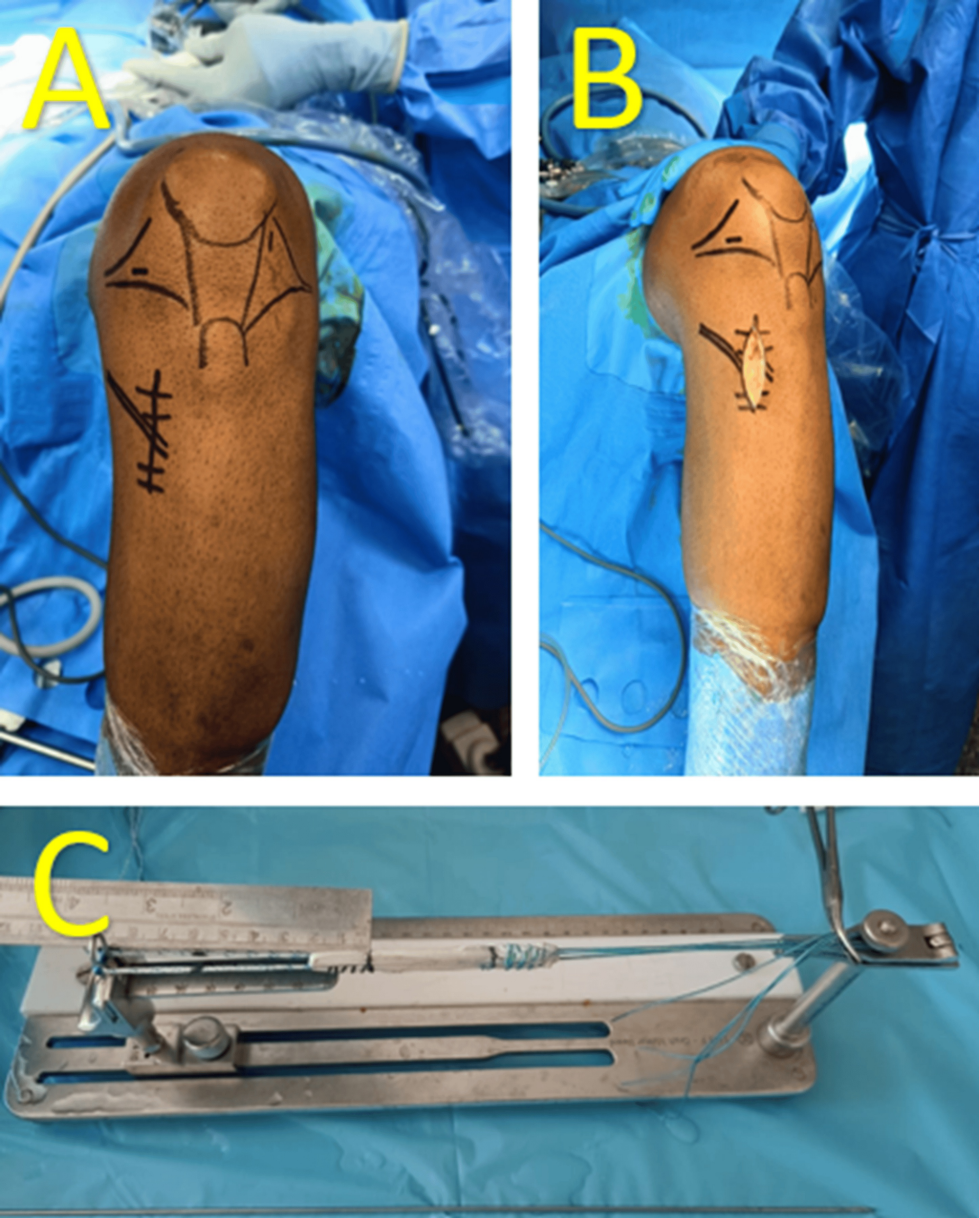
Intraoperative picture (A) Intraoperative picture showing skin marking, (B) graft harvesting site, and (C) harvested semitendinosus graft loaded on Endobutton.

All patients underwent standard ACL reconstruction surgical steps (Figure 1), including portal creation (anterolateral and anteromedial portals), graft harvesting (semitendinosus tendon with optional gracilis graft), graft preparation (measuring, suturing, and passing through an adjustable loop Endobutton to create a four-strand quadrupled hamstring graft), ACL stump removal and notch preparation, tunnel drilling (femoral and tibial tunnels), and graft passage and fixation (femoral tunnel passage and tibial fixation using a 30 mm bioscrew with a diameter 1 mm larger than the graft diameter). 

Supplementary tibial fixation

**Figure 2:**
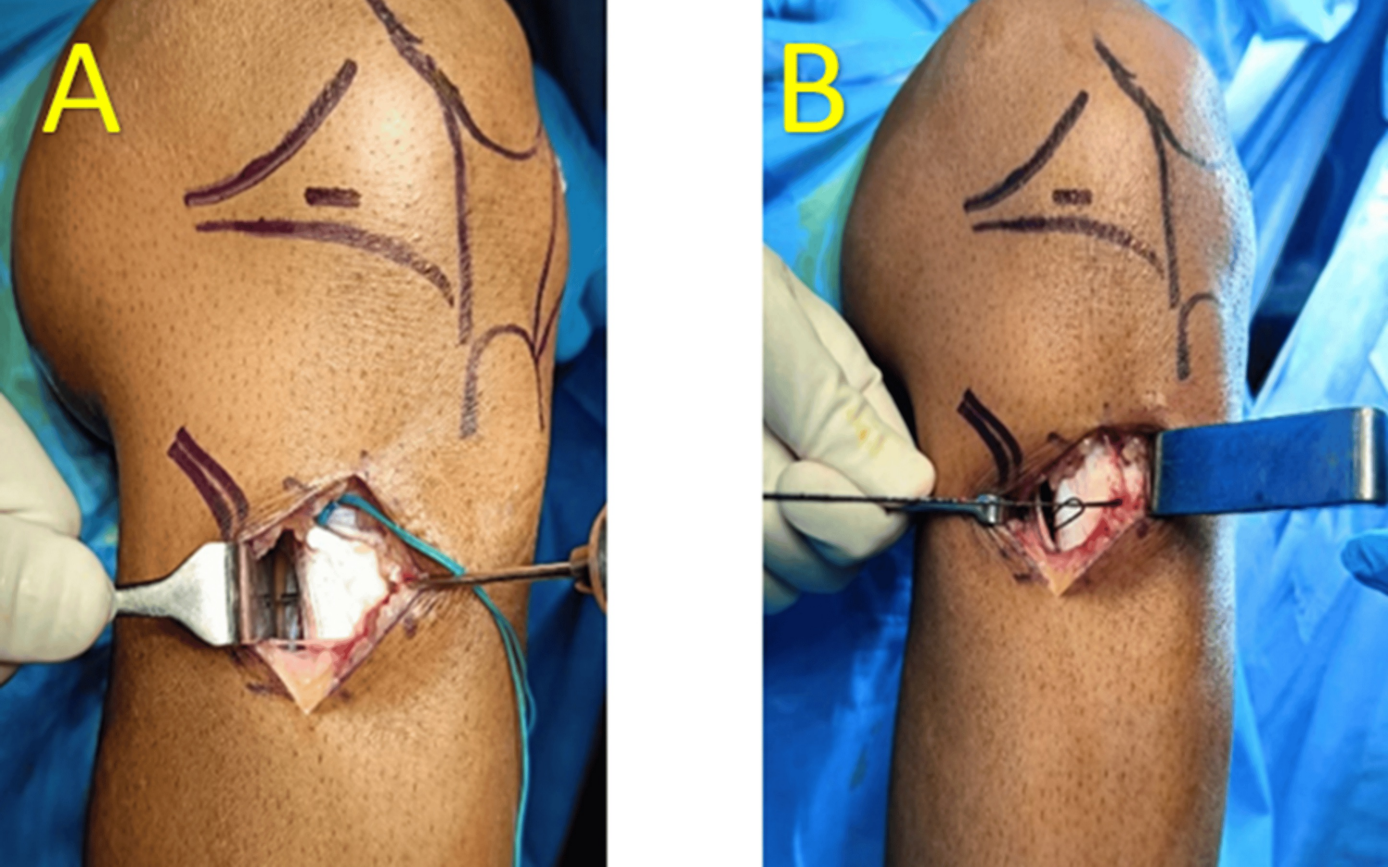
(A) Tibia tunnel made through K-wire, (B) 1-0 Vicryl suture passed and loop made using beath pin

**Figure 3:**
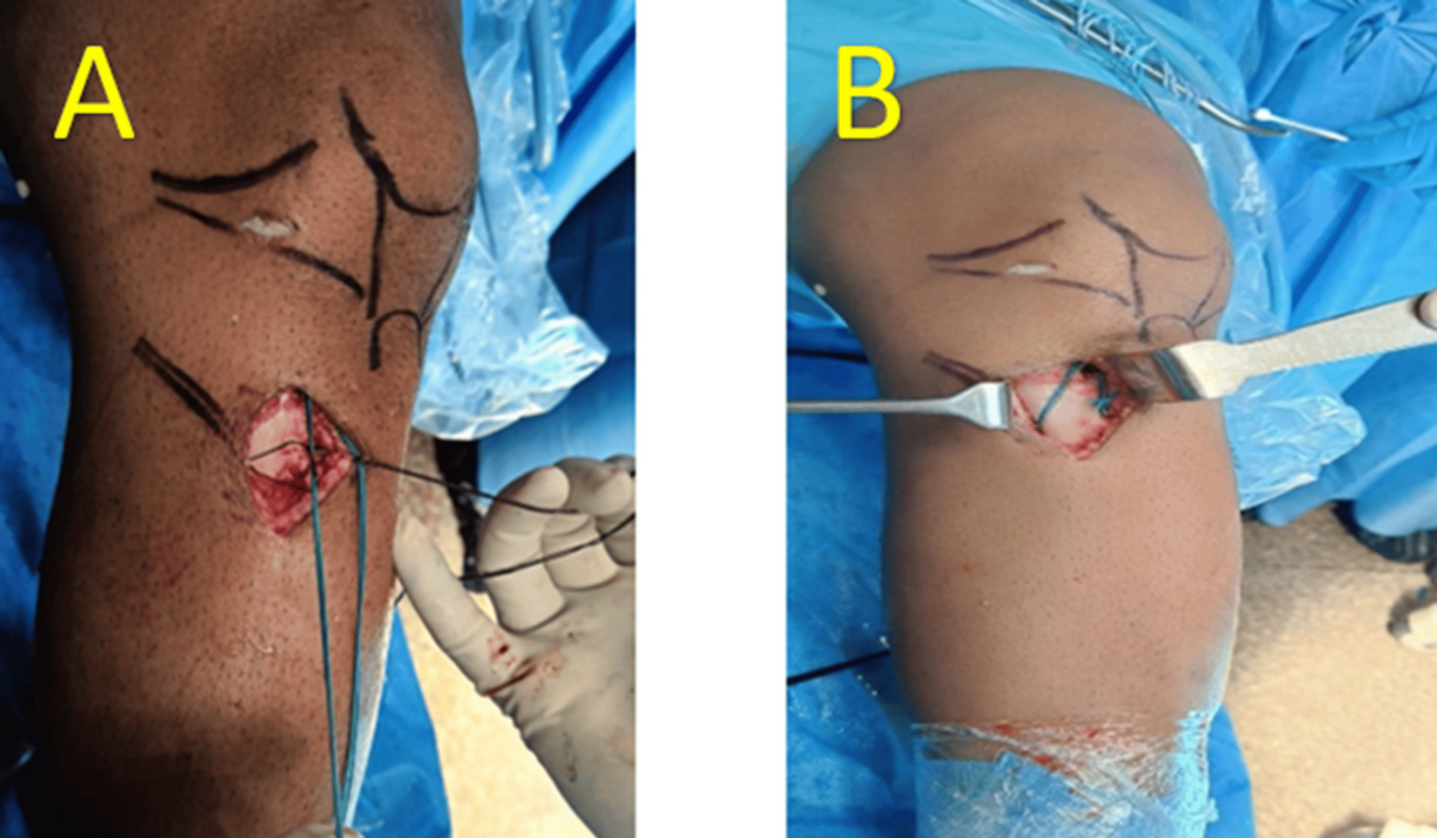
(A) No. 5 Ethibond suture attached to graft shuttled through the tibial tunnel made, (B) Ethibond ends tied and fixation secured

**Figure 4:**
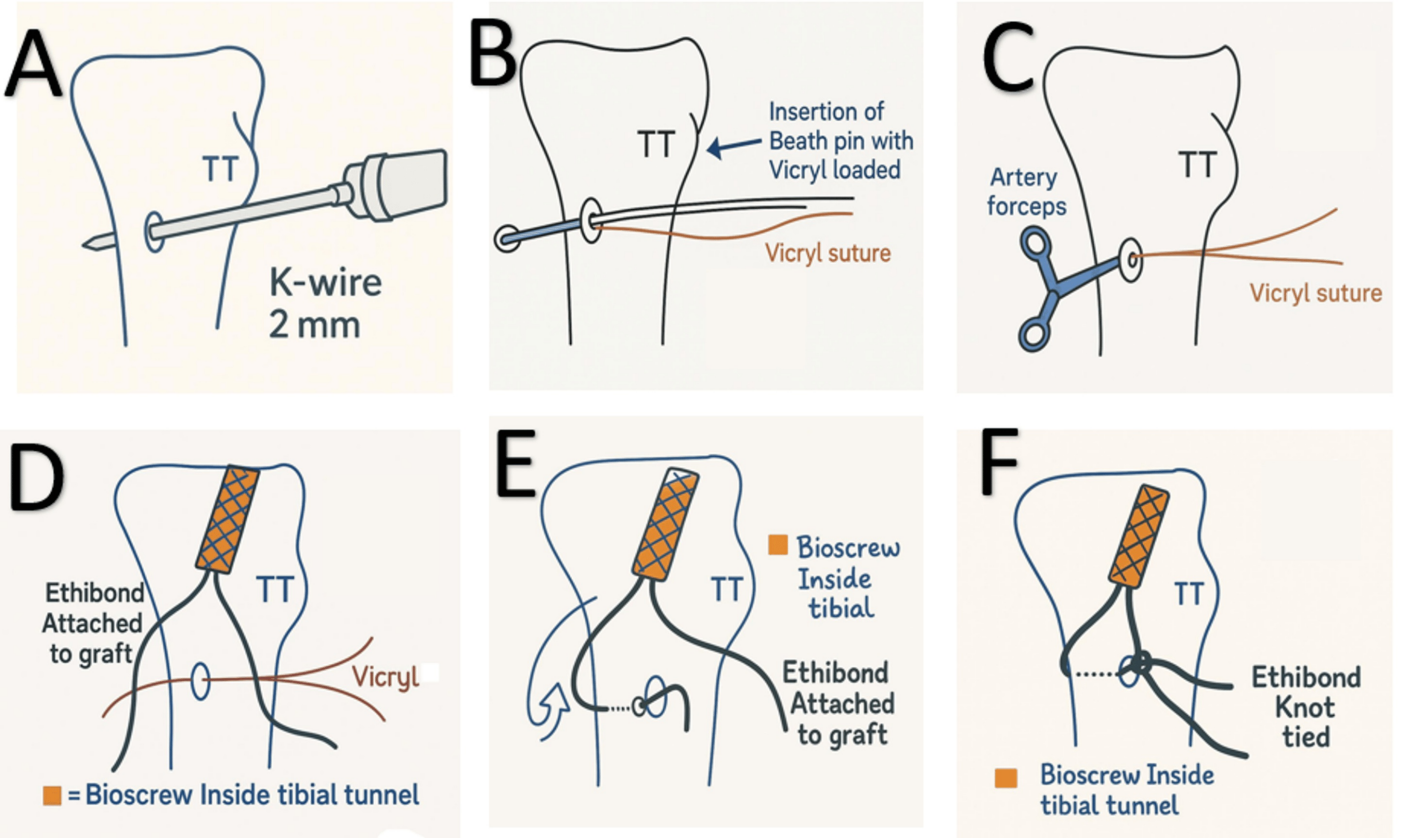
Line diagram of surgical steps for supplementary transosseous tibial fixation (A) Tibial tunnel made at the hamstring insertion site using a 2-mm K-wire. (B) 1-0 Vicryl suture inserted using a beath pin. (C) Vicryl suture loop held with artery forceps. (D) No. 5 Ethibond threads of the hamstring graft passed through the Vicryl suture loop. (E) One of the Ethibond ends brought anteriorly. (F) Ethibond threads tied using simple and reverse knots. Figure credits: Created by the authors.

The steps of supplementary fixation (Figures 2,3), as shown in Figure 4, include creating a tibial tunnel at the hamstring insertion site using a 2 mm K-wire, drilling in an anteroposterior direction, and making a small window in the fascia at the posteromedial border of the tibia. A 1-0 Vicryl suture is then inserted through the hole using a straight needle, and the suture loop is brought anteriorly using curved artery forceps. Two number 5 Ethibond (Ethicon, Cincinnati, USA) threads of the hamstring graft are passed through the Vicryl (Ethicon, Cincinnati, USA) suture loop, which is then pulled anteriorly, bringing both threads anteriorly. Finally, the threads are tied to the remaining graft threads using simple and reverse knots, and the remaining threads are cut.

Post-op management

All patients underwent a standardized rehabilitation program after ACL reconstruction. A knee brace was applied immediately, and quadriceps exercises were initiated. Daily physical therapy sessions targeted knee extension and muscle contractions. Patients progressed through a tailored rehabilitation program with regular follow-ups to monitor progress. The goal was to return patients to sporting activities within 6-9 months.

Assessment

Patients underwent examination pre-operatively (baseline), and at 12 months and 24 months post-operatively. Clinical and functional assessment was done using various outcome measures, including Lachman test, Pivot test, Lysholm knee score, and IKDC score at the 2-year follow-up. Lysholm knee score (range: 0-100) is a self-administered questionnaire evaluating knee function, including symptoms such as limp, need for support, locking, instability, pain, swelling, and impairment of stair climbing or the ability to squat. IKDC (International Knee Documentation Committee) score (range: 0-100) assesses knee function and symptoms, including pain, stiffness, swelling, locking, giving way, pivot shift, Lachman test, and functional ability, with higher scores indicating better knee function and fewer symptoms. Clinical Assessment was done using the Lachman test (grading: 0-3), evaluating knee stability, with grade 0 indicating <2 mm, grade 1 indicating 3-5 mm, grade 2 indicating 6-9 mm, and grade 3 indicating >10 mm compared to the contralateral side. Integrity and laxity were also assessed using the pivot shift test at all follow-ups, with grade 0 indicating no shift, grade 1 indicating a glide or a feeling of a subtle shift, grade 2 indicating the tibia reducing with a clunk, and grade 3 indicating that the tibia looked in a subluxated position. Patients were evaluated for kneeling pain and hamstring discomfort using a Visual Analog Scale (VAS) at pre-op, 1-year, and 2-year follow-ups. The VAS rated pain from 0 (no pain) to 100 (most severe pain). Thigh atrophy was assessed at the 2-year follow-up by measuring the circumference 10 cm above the patella on both legs, with a difference of less than 1 cm being considered normal and more than 1 cm as significantly atrophied.

Statistical analysis

The collected data were systematically entered into Excel sheets (Microsoft Corporation, Redmond, USA) and subsequently analyzed using SPSS software (IBM Corp., Armonk, USA). Pre-operative and post-operative data were compared using paired t-tests. For comparisons between the two cohorts, unpaired t-tests were employed for continuous data, while chi-square tests were used for categorical data. Statistical significance was set at p < 0.05.

## Results

A total of 88 patients were available for follow-up evaluation two years post-surgery. The mean age of the patients in the standard group was 30.3 years, ranging from 19 to 45 years, whereas the supplementary fixation group had a mean age of 30.9 years, with an age range of 21 to 42 years. In our study, ACL injuries primarily occurred due to sports activities (62.5%), followed by road traffic accidents (28.4%), and other mechanisms (9.1%). Athletes and individuals with physically demanding jobs were more commonly affected. The average follow-up duration was 33.9 and 34.3 months, respectively. The timing of ACL reconstruction varied, with 35 patients undergoing surgery in the subacute phase (3-12 weeks post-injury) and 53 patients in the chronic phase (>12 weeks post-injury). Mean femoral tunnel diameter in both groups was found to be the same at 7.5 mm.

The two groups were matched in terms of age, side, chronicity, and follow-up duration (Table 1). Additionally, there were no statistically significant differences between the two groups in pre-operative Lysholm knee scores (Table 2), ensuring that the two groups were comparable at baseline, allowing a fair comparison of the outcomes.

**Table 1:** Comparison of the baseline parameters between the standard and supplementary groups Groups data given as frequency (n) or mean ± SD. Chronicity refers to the time elapsed between the injury and the surgery. Unpaired t-test applied. Z-test for two-sample proportion applied. NS: not significant

	Standard Group (n=40)	Supplementary Fixation Group (n=48)	t-value/Z-value	Significance (p-value)
Mean Age	30.3 ± 6.15 yrs (19-45)	30.9 ± 5.57 yrs (21-42)	0.4799, df=86	0.63, NS
Average Follow-Up	33.90 ± 1.30 months	34.3 ± 2.67 months	0.8653, df=86	0.389, NS
Side				
Left	18	22	Z value= -0.0782	0.936, NS
Right	22	26	Z value= 0.0782	0.936, NS
Chronicity				
Subacute (3-12 Weeks)	15	20	Z value= -0.3977	0.689, NS
Chronic (>12 Weeks)	25	28	Z value= 0.3977	0.689, NS

**Table 2:** Comparison of the mean Lysholm knee scores and thigh atrophy between the groups at 2 years follow-up. *Significant; NS: not significant. Paired t-test for within-group comparison, unpaired t-test for between-groups comparison, and Z-test for two-sample proportion for proportional comparison.

	Standard Group	Supplementary Group	t-value, df	Significance (p-value)
Preoperative Lysholm Score, mean ± SD	67.05 ± 2.97	66.19 ± 1.45	1.771, df=86	0.080, NS
Post-operative Lysholm Score, mean ± SD	84.95 ± 0.71	94.56 ± 1.53	-36.560, df=86	0.001*
Pre- and Post-operative Score Comparison	-36.758, df=39	-95.961, df=47		
Significance	0.001*	0.001*		
Thigh Atrophy (less than 1 cm side-to-side difference) (n (%))	10 (25%)	20 (42%)	Z value= -1.6424	0.101, NS

We observed significant improvements in functional knee scores post-operatively in both groups, with significantly better results in the supplementary fixation group as compared to the standard group (Table 2). At the 2-year follow-up, 95% and 98% of patients in standard and supplementation groups, respectively, revealed good to excellent Lysholm knee scores (Table 3), among which, the supplementation group had a higher score of 94.56 (86-95) as compared to the standard group, which scored 84.95 (79-87), with post operative Lysholm score results comparison being significant (p=0.001). Patients in the supplementary group tended to have less muscle atrophy at 2-year follow-up as compared to the contralateral side group, with 42% (20 patients) exhibiting a thigh circumference difference of less than 1 cm in the supplementary group, compared to 25% (10 patients) in the standard group (p = 0.101).

**Table 3:** Comparison of Lysholm knee grades between the groups at the 2-year follow-up (Pearson chi-square test applied). NS: not significant. Groups data given as n (%).

Lysholm Knee Score	Standard Group n =40	Supplementary Group n=48	Chi-square Value, df	p-value
Excellent	21 (52.5%)	29 (60.4%)	0.922, df=2	0.630, NS
Good	17 (42.5%)	18 (37.5%)		
Fair	2 (5%)	1 (2.1%)		
Poor	0	0		

Lachman and pivot tests

Manual ligament examination was done using the Lachman and pivot tests at the 2-year follow-up. Overall, 75% and 86% of patients (n=88) showed zero results in the Lachman and pivot tests, respectively, at the 2-year follow-up. Although the results of the Lachman test showed an improvement in both the supplementary and standard groups, the supplementary group had significant results for grade 0 and grade 1 Lachman grading at the 2-year follow-up (p = 0.038). Similarly, the supplementary group performed better in the pivot test, but the results were not significant (p =0.334) (Table 4).

**Table 4:** Comparison of the Lachman and pivot shift test grades at the 2-year follow-up for the two groups (Pearson chi-square test applied). *Significant; NS: not significant. Groups data given as n (%)

Test	Standard Group (n=40)	Supplementary Fixation Group (n=48)	Chi-square Value, df	p-value
Lachman			6.539, df=2	0.038*
Grade 0	25 (62.5%)	41 (41.7%)		
Grade 1	14 (35%)	07 (14.6%)		
Grade 2	01 (2.5%)	0		
Grade 3	0	0		
Pivot shift			0.93, df=1	0.334, NS
Grade 0	33 (82.5%)	43 (89.5%)		
Grade 1	07 (17.5%)	05 (10.5%)		
Grade 2	0	0		
Grade 3	0	0		

Visual Analog Score (VAS) for pain assessment

Visual analog score (VAS Score) was used to assess kneeling pain at the 2-year follow-up. Nearly all the patients in both groups had none to mild pain except for one patient in the standard group found to have moderate pain, with results being non-significant (p=0.369) (Table 5).

**Table 5:** Comparison of VAS for the two groups at the 2-year follow-up (Pearson chi-square test applied). NS: not significant; VAS: Visual Analog Score

VAS score grade	Standard Group, n	Supplementary Group, n	Chi-square value, df	P value
No Pain (0)	25	35	1.993, df=2	0.369, NS
Mild (1-30)	14	13		
Moderate (31-60)	1	0		
Severe (61-90)	0	0		
Worst (91-100)	0	0		

International Knee Documentation Committee (IKDC) grade assessment

Most patients in both groups achieved normal or nearly normal results: 82.5% in the standard group and 89.5% in the supplementary group (Table 6). Subjective assessments showed high levels of recovery and functional restoration. Symptoms assessment revealed no statistically significant differences between the two groups regarding pain, swelling, or episodes of giving way (partial or full) during moderate to strenuous activities (p = 0.600). No patients exhibited significant extension or flexion losses (>3° or >5°, respectively). Although the activity levels were similar in both groups, the supplementary fixation group performed better, with 63% of the group participating in level 1 (pivot, jump, cutting) and level 2 (including heavy manual work) activities as compared to 42% in the standard group.

**Table 6:** Comparison of the IKDC grades at the 2-year follow-up for both groups (Pearson chi-square test applied). IKDC: International Knee Documentation Committee; NS: not significant. Groups data given as frequency (n).

IKDC Grade	Standard Group (n=40)	Supplementary Group (n=48)	Chi-square value, df	p-value
Grade A-Normal Knee	10	15	1.869, df=3	0.600, NS
Grade B-Near Normal	23	28		
Grade C-Abnormal Knee	6	5		
Grade D-Severely Abnormal	1	0		

## Discussion

Arthroscopic ACL reconstruction with hamstring graft is a common procedure, but there's no consensus on supplementary tibial fixation. Our technique offers a less expensive option without symptomatic hardware risks. We compared ACL reconstruction results using a quadrupled semitendinosus graft with an adjustable loop Endobutton and bioscrew, with or without supplementary tibial fixation using a tibial bone tunnel.

Our study results demonstrate that with the use of supplementary tibial fixation along with bioscrew fixation for ACL reconstruction using quadrupled semitendinosus graft, there is significant improvement in the Lachman test and Lysholm score at the 2-year follow-up as compared to the standard group, with the former performing better in IKDC scoring. Similar results were also found in a study conducted by Hill et al. [13] using a hamstring graft in female patients with supplementary fixation using a staple, however, at the cost of increased kneeling pain due to symptomatic hardware.

Kneeling pain could be troublesome for patients with kneeling as part of their occupation and religious concerns. Previous studies have also investigated post-ACL reconstruction complications. Bak et al. [14] reported that 15% of patients experienced discomfort after ACL reconstruction using an iliotibial band autograft, with 25% requiring staple removal. Similarly, Billotti et al. [15] found that 7% of patients underwent staple removal due to localized pain after ACL reconstruction using the semitendinosus and iliotibial band. Our study eliminates this drawback as no new implant was used in our study, at the same time being cost-effective.

Our study's technique for supplementary tibial fixation in ACL ligament reconstruction using hamstring tendon graft with transosseous sutures is consistent with a similar procedure described by Leyes et al [16] using bone-tendon-bone graft. The similarity between our study's methodology and that of Leyes et al. [16] provides validation for the effectiveness of this technique in enhancing ACL reconstruction outcomes.

Lim et al. [17] conducted a similar study comparing fixation of a hamstring graft using bone staples for supplementary fixation in male patients undergoing arthroscopic ACL reconstruction and found significantly better outcomes in terms of instrumented ligament testing and subjective knee scores in the staple group. Madadi et al. [18] also reported superior results using supplementary fixation of a hamstring graft, with more patients achieving the goal of <3 mm anterior translation of tibia on arthrometer testing as compared to patients with bio-interference screw fixation alone. Our study had similar findings with significant differences in the Lachman and Lysholm scores of patients who underwent ACL reconstruction with supplementary fixation as compared to those with ACL reconstruction alone.

De Wall et al. [19] conducted a randomized controlled trial on patients undergoing ACL reconstruction using multiple staples and found no difference in the Lysholm scores, IKDC, or laxity testing. Studies by Noh et al. [20] and Simonian et al. [21] comparing ACL reconstruction with or without supplementary fixation also found no difference in subjective scores and laxity testing. Superior results with supplementary tibial fixation as found in our study may be explained using a relatively large sample size, methodology avoiding bias, and no extra implant, avoiding any hardware impingement on extreme flexion.

Our study demonstrated that enhanced stability correlated with clinically significant improvements, along with reduced thigh atrophy and improved Visual Analog Scale (VAS) scores at the 2-year follow-up. Similar findings were found in a study conducted by Lim et al. [17], thus validating our results. A possible explanation for this could be the enhanced stability provided by the additional fixation. This stability may promote better graft integration, reducing stress on surrounding tissues, leading to improved knee function. As a result, patients may experience increased muscle activity, reduced atrophy, and decreased pain. The enhanced stability and improved knee function likely contribute to the observed benefits in thigh atrophy and pain reduction. Alternative methods for double tibial fixation, such as using screws and anchors, are available. However, these alternatives come with increased costs and heightened risks, including symptomatic hardware complications and mechanical failure.

There have been studies that have demonstrated that during ACL reconstruction, the tibia is the weakest point of fixation as it has less dense bone compared to the femur, and the force vector lies parallel to the tibial tunnel as compared to the femur [22]. Trojani et al. [23] studied failed ACL reconstruction cases and found that out of a total of 293 failed ACL cases, 5% were early failures before graft incorporation, likely due to graft slippage.

Another factor to be taken into consideration is the impact of ACL Injury on bone mineral density. ACL injury has been shown to lead to decreased bone mineral density, both in the acute and chronic phases [24,25]. Studies have found that operatively treated ACL ruptures experience a significantly greater reduction in bone mineral density compared to conservatively treated cases [26]. This decrease in bone mineral density can compromise the stability of ACL reconstruction.

Hence providing additional fixation in the tibia helps in reducing postoperative laxity and better subjective scores as compared to standard biointerference screw fixation alone. Furthermore, studies [27] have consistently shown that Asian populations tend to have lower bone mineral density compared to other ethnic groups. This inherent difference further supports the importance of supplementary fixation techniques to ensure optimal outcomes and minimize the risk of fixation failure in this demographic.

Limitations of the study

The study had several limitations, including limited statistical power due to small sample size, lack of biomechanical studies and high-level evidence, inclusion of both sexes despite non-significant sex distribution, short study duration, and limited generalizability. Also, the study did not compare bone mineral density between the two groups, which could potentially impact the outcomes of ACL reconstruction. Although the age and sex distribution were comparable between the groups with non-significant differences, thus minimizing potential bias, future studies would benefit from bone mineral density comparisons to further elucidate its impact on outcomes. These limitations may have masked potential differences between groups. Despite this, the technique has shown promising results in over 150 cases, with improved clinical and functional outcomes and no major complications. Further studies with larger sample sizes and longer durations are necessary to validate the findings.

## Conclusions

Supplementary fixation with trans osseous tibial tunnel is a safe, effective, and cost-efficient method for improving knee stability and clinical outcomes. Our study found Significantly better Lachman and Lysholm scores along with better pivot shift and IKDC scores and Improved Visual Analog Scale (VAS) scores at 2-year follow-up. This study also eliminates the risk of symptomatic hardware complications as was found in several previous studies and avoids the need for extra implants, reducing overall treatment costs

## References

[1] Ahldén M, Samuelsson K, Sernert N, Forssblad M, Karlsson J, Kartus J (2012). The Swedish National Anterior Cruciate Ligament Register: a report on baseline variables and outcomes of surgery for almost 18,000 patients. Am J Sports Med.

[2] Goldblatt JP, Fitzsimmons SE, Balk E, Richmond JC (2005). Reconstruction of the anterior cruciate ligament: meta-analysis of patellar tendon versus hamstring tendon autograft. Arthroscopy.

[3] Wagner M, Kääb MJ, Schallock J, Haas NP, Weiler A (2005). Hamstring tendon versus patellar tendon anterior cruciate ligament reconstruction using biodegradable interference fit fixation: a prospective matched-group analysis. Am J Sports Med.

[4] Li S, Chen Y, Lin Z, Cui W, Zhao J, Su W (2012). A systematic review of randomized controlled clinical trials comparing hamstring autografts versus bone-patellar tendon-bone autografts for the reconstruction of the anterior cruciate ligament. Arch Orthop Trauma Surg.

[5] Nakamura N, Horibe S, Sasaki S (2002). Evaluation of active knee flexion and hamstring strength after anterior cruciate ligament reconstruction using hamstring tendons. Arthroscopy.

[6] Gobbi A (2010). Single versus double hamstring tendon harvest for ACL reconstruction. Sports Med Arthrosc Rev.

[7] Aggarwal P, Mahapatra S, Awasthi S, Kumar V (2020). Functional outcomes of quadrupled semitendinosus vs. four strand semitendinosus / Gracilis graft for arthroscopic ACL reconstruction. Int J Orthop Sci.

[8] Tomita F, Yasuda K, Mikami S, Sakai T, Yamazaki S, Tohyama H (2001). Comparisons of intraosseous graft healing between the doubled flexor tendon graft and the bone-patellar tendon-bone graft in anterior cruciate ligament reconstruction. Arthroscopy.

[9] Kohn D, Rose C (1994). Primary stability of interference screw fixation. Influence of screw diameter and insertion torque. Am J Sports Med.

[10] Colombet P, Graveleau N, Jambou S (2016). Incorporation of hamstring grafts within the tibial tunnel after anterior cruciate ligament reconstruction: magnetic resonance imaging of suspensory fixation versus interference screws. Am J Sports Med.

[11] Ekdahl M, Wang JH, Ronga M, Fu FH (2008). Graft healing in anterior cruciate ligament reconstruction. Knee Surg Sports Traumatol Arthrosc.

[12] Balazs GC, Brelin AM, Grimm PD, Dickens JF, Keblish DJ, Rue JH (2016). Hybrid tibia fixation of soft tissue grafts in anterior cruciate ligament reconstruction: a systematic review. Am J Sports Med.

[13] Hill PF, Russell VJ, Salmon LJ, Pinczewski LA (2005). The influence of supplementary tibial fixation on laxity measurements after anterior cruciate ligament reconstruction with hamstring tendons in female patients. Am J Sports Med.

[14] Bak K, Jørgensen U, Ekstrand J, Scavenius M (1999). Results of reconstruction of acute ruptures of the anterior cruciate ligament with an iliotibial band autograft. Knee Surg Sports Traumatol Arthrosc.

[15] Billotti JD, Meese MA, Alberta F, Zimmerman MC (1997). A prospective, clinical study evaluating arthroscopic ACL reconstruction using the semitendinosus and iliotibial band: 2- to 5-year follow up. Orthopedics.

[16] Leyes M, González-Martín D, Flores-Lozano C, González-Salvador M, Martín-Buenadicha E, García-Crespo R (2022). Supplementary tibial fixation in anterior cruciate ligament reconstruction with bone-tendon-bone graft. Arthrosc Tech.

[17] Lim CT, Tan KJ, Chuan AK (2009). Clinical stability and outcome of supple- menting tibial fixation with a staple for ACL reconstruction using hamstring tendons. Curr Orthop Pract.

[18] Madadi F, Sarmadi A, Kahlaee AH (2010). A new hybrid fixation method in ACL reconstruction surgery. Eur J Orthop Surg Traumatol.

[19] De Wall M, Scholes CJ, Patel S, Coolican MR, Parker DA (2011). Tibial fixation in anterior cruciate ligament reconstruction: a prospective randomized study comparing metal interference screw and staples with a centrally placed polyethylene screw and sheath. Am J Sports Med.

[20] Noh JH, Yang BG, Yi SR, Roh YH, Lee JS (2012). Hybrid tibial fixation for anterior cruciate ligament reconstruction with Achilles tendon allograft. Arthroscopy.

[21] Simonian PT, Monson JT, Larson RV (2001). Biodegradable interference screw augmentation reduces tunnel expansion after ACL reconstruction. Am J Knee Surg.

[22] Brand J Jr, Weiler A, Caborn DN, Brown CH Jr, Johnson DL (2000). Graft fixation in cruciate ligament reconstruction. Am J Sports Med.

[23] Trojani C, Sbihi A, Djian P (2011). Causes for failure of ACL reconstruction and influence of meniscectomies after revision. Knee Surg Sports Traumatol Arthrosc.

[24] Bayar A, Sarikaya S, Keser S, Ozdolap S, Tuncay I, Ege A (2008). Regional bone density changes in anterior cruciate ligament deficient knees: a DEXA study. Knee.

[25] Kannus P, Sievänen H, Järvinen M, Heinonen A, Oja P, Vuori I (1992). A cruciate ligament injury produces considerable, permanent osteoporosis in the affected knee. J Bone Miner Res.

[26] Boyd SK, Matyas JR, Wohl GR, Kantzas A, Zernicke RF (2000). Early regional adaptation of periarticular bone mineral density after anterior cruciate ligament injury. J Appl Physiol (1985).

[27] Tobias JH, Cook DG, Chambers TJ, Dalzell N (1994). A comparison of bone mineral density between Caucasian, Asian and Afro-Caribbean women. Clin Sci (Lond).

